# The economic burden of nosocomial infections for hospitals: evidence from Germany

**DOI:** 10.1186/s12879-024-10176-8

**Published:** 2024-11-13

**Authors:** Lulseged M. Asegu, Anne Kitschen, Meike M. Neuwirth, Dirk Sauerland

**Affiliations:** 1 Witten/Herdecke University, Department of Philosophy, Politics and Economics, Chair for Institutional Economics and Health Policy, Witten, Germany; 2 Witten/Herdecke University, Division of Hygiene and Environmental Medicine, Cologne, Germany; 3 Institute for Hygiene, Cologne Merheim Medical Centre, University Hospital Witten/Herdecke, Cologne, Germany; 4 Witten/Herdecke University, Interdisciplinary Centre for Health Services Research, Witten, Germany

**Keywords:** Nosocomial Infection, Opportunity Costs, Economic Burden, Hospitals, Genetic Matching

## Abstract

**Background:**

Nosocomial infections (NI) significantly worsen patient outcomes, resulting in higher mortality rates and reduced health-related quality of life. Furthermore, they pose substantial economic strain on healthcare systems and hospitals. For instance, patients with nosocomial infections (NIs) experience prolonged hospital stays compared to those without NIs. These extended stays result in occupied bed-days, leading to opportunity costs for hospitals. This study aimed to estimate the opportunity costs for a German hospital based on hospital stays, daily revenue, and occupancy rates (OCR).

**Methods:**

We analysed cost data obtained from routine records maintained by the accounting department of a German hospital's surgical and orthopedic units from 2018 to 2019 for the “HygArzt” research project. To ensure balance, we employed genetic matching. We estimated the differences in length of stay (LOS) and daily revenue between patients with and without nosocomial infections (NI) using linear regression. Finally, we calculated the opportunity cost borne by the hospital by treating NI patients instead of non-NI patients. All costs are reported in 2018 Euros.

**Results:**

The final sample included 81 patients with NI matched with 207 patients without NI. The majority of the NI patients (77.0%) had surgical site infection (SSI). Compared to non-NI patients, we observed that NI patients had a longer LOS (10 days, *p* < 0.001) and lower daily revenue (€400, *p* < 0.001). We also found that comorbidities and the frequency of operations had significant impact on the LOS. Using a baseline 30 to 50% preventable NIs, successful prevention of a single NI could potentially reduce the length of hospital stay by 3 to 5 days and increase hospital revenue by approximately €120 to €200 per day per prevented NI. Consequently, the hospital saves 3 to 5 more bed-days to backfill and generate more revenue, and/or make more efficient resource allocation by changing bed-capacity and staffing. The resulting opportunity costs can potentially exceed €1,000 per preventable case.

**Conclusion:**

NIs pose a substantial economic burden for hospitals. From a health economics’ perspective, there are strong economic incentives for hospitals to implement infection control interventions, such as the involvement of a prevention link physician/nurse.

**Supplementary Information:**

The online version contains supplementary material available at 10.1186/s12879-024-10176-8.

## Background

Nosocomial infections (NIs), also known as hospital-acquired infections, are defined by the World Health Organization as infections that are acquired during hospital care, are not present, or are incubated at admission [1]. Infections occurring more than 48 hours after admission are considered NI [1]. The prevalence of NI was 5.9% from 2016 to 2017 in the European Union and 4.6% in Germany [2]. The most common NIs were respiratory tract infections (pneumonia 21.4% and lower respiratory tract infections 4.3%), urinary tract infections (18.9%), surgical site infections (SSI) (18.4%), bloodstream infections (10.8%), and gastrointestinal infections (8.9%), including Clostridium difficile-associated diarrhea (CDAD) [3].

The clinical burden of NIs is primarily related to their impact on patient health and the challenges they pose to the healthcare delivery process. High infection rates contribute to increased morbidity and mortality among patients. Prolonged hospital stays, heightened susceptibility to complications, and the demand for additional medical interventions are key indicators of clinical burden. NIs not only compromise patient well-being but also strain healthcare resources, leading to challenges in managing and treating affected individuals. Several systematic reviews have analysed the clinical burden of NIs. For example, the estimated mortality rate of a bloodstream infection ranges between 21.6 and 37.8 per 100,000 person-years in Europe [4]. The short-term case fatality rate following bloodstream infection is 13% to 20% in Europe [4]. Additionally, patients with hospital-acquired multidrug-resistant *Pseudomonas aeruginosa* have increased all-cause mortality [5]. The same applies to nosocomial pneumonia: critically ill patients with ventilator-associated pneumonia are twice as likely to die as similarly ill patients without ventilator-associated pneumonia [6]. Apart from their physical impact, NIs may have an effect on health-related quality of life. Avsar et al. [7] reported a lower quality of life with limitations in physical, social and psychological functioning in patients experiencing a surgical site infection [7].

In addition to these serious impacts on patients’ health, NIs are also associated with a significant economic burden [8]. The economic burden associated with a disease is commonly analysed from a societal, healthcare payer, or healthcare provider perspective. From a societal perspective, all disease-related costs are relevant and should be considered in the analysis, including the associated productivity loss caused by NIs. From a healthcare payer perspective, all costs that are reimbursed by the payer are relevant, e.g., outpatient and inpatient services. The perspective of the healthcare provider includes costs that must be covered by the provider itself.

In the case of NI, it is crucial to consider the hospitals’ perspective, as their costs determine the incentives to implement infection prevention measures. Looking at the relevant costs in more detail, opportunity costs play a major role from the hospital perspective. These costs primarily arise from a longer length of stay [9, 10] for patients with NI, which results in blocked beds either due to a longer stay or due to isolation. Bed blockages prevent new patients from being admitted, resulting in lost revenues for the hospital [8, 11–13]. The number of studies analyzing opportunity costs from a hospital perspective in Germany is limited. For example, a retrospective study focusing solely on CDAD estimated an opportunity cost of €5,262.96 per case. Notably, these costs constituted 94.4% of the overall hospital expenses [8]. The objective of this study is to estimate the opportunity costs of NIs for a German hospital. This will help to explore the potential impact of infection prevention strategies.

## Methods

### Data description

This non-experimental study (“HygArzt” research project)[14] utilizes anonymized data generated during standard medical practice, collected in compliance with HygmedVoNRW §8 and the Infection Protection Law §23, and protected according to the Federal Data Protection Act (BDSG), General Data Protection Regulation (GDPR), and the professional code of conduct for physicians in North Rhine-Westphalia [15–17].

NIs were defined following the Hospital Infection Surveillance System (KISS), which is based on the US National Healthcare Safety Network (NHSN) guidelines by the Centers for CDC [18, 19]. An infection is classified as nosocomial if it occurs within 90 days of surgery, or after the last surgery in the case of consecutive procedures, following KISS definitions for implants[18].Only infections developing more than 48 hours post-admission or after surgery in the orthopedic unit are included [14]. Patients who left the hospital against medical advice, infections acquired prior to the study and those occurring in intensive care units or within 48 hours of admission are excluded, and recurrent infections of the same type are recorded only once [14].

The available routine data includes details on Diagnosis-Related Groups (DRG), types of NIs (SSI, CDAD, UTI, pneumonia, bronchitis, BSI, and thrombophlebitis), and patient information such as age, sex, comorbidities, frequency of operations, ICD and OPS coding, and DRG revenue per patient. While several representative German hospitals provide anonymized datasets—including detailed cost data—to the Institute for Hospital Remuneration (InEK) [20], which contain both standard DRG payments and patient-specific direct variable costs for broader research [21, 22], our dataset comes from routine records of a hospital’s accounting department provided for a single hospital unit. As a result, we focus on revenues since direct variable costs were not captured.

### Theoretical framework

The economic assessment of successful NI prevention in hospitals hinges on discerning the treatment costs for NI patients vis-à-vis regular cases. This differentiation is crucial for delineating direct and indirect costs, as well as fixed and variable costs within healthcare expenditures.

The specific antibiotics required for NI patient treatment influence direct costs and are quantifiable. As the number of NI cases affect these variable costs, their analysis proves essential; a decrease in NI instances results in a corresponding reduction in variable direct treatment expenses. Conversely, the assessment of NI prevention benefits does not necessitate consideration of direct fixed treatment costs, which include hospital expenses such as personnel, beds, buildings, and equipment, which are crucial for treatment capacity and remain unaffected by patient numbers or NI cases (at least for a short period of time).

Indirect costs are incurred within the hospital's treatment capacity due to missed opportunities resulting from the treatment of NI patients. Successful prevention measures would free up capacity, allowing for the treatment of additional "normal cases" within existing limits, potentially increasing revenue. This scenario, where fixed costs remain constant but revenues increase, offers the hospital an opportunity to enhance profitability.

Assessing the direct costs of NIs presents challenges due to the hospital's cost accounting practices, where pharmaceuticals and treatments are typically not allocated to individual patients. Instead, data are aggregated at the department level, making it difficult to attribute to specific individuals. Hence, our analysis focused on evaluating the indirect opportunity costs associated with bed days allocated to NI patients.

Based on the available data, we calculate the opportunity costs of bed days (OC) of NI patients as the difference between the total revenues foregone (TRF) and the total revenues realised (TRR) for the hospital.

1 $$OC=TRF-TRR$$

To compute the total revenues foregone (TRF), we multiply the average length of stay (LOS) for NI patients by the realised revenues per day (DRR) for "normal cases" and adjust for the typical occupancy rate (OCR). This approach, discussed in Sandman et al. (2018), allows us to estimate the potential revenues lost by treating NI patients instead of "normal cases" within the existing capacity (Equation 1).

2 $${TRF}_{NI}={LOS}_{NI}*{DRR}_{Normal}*OCR$$

However, hospitals also realize revenues for the NI patients they treat. Therefore, we also analyse the revenues realized (TRR) by treating patients with NI. We compute the TRR in equation (3) as contingent on the daily revenues realized (DRR) and LOS of NI patients.

3 $${TRR}_{NI}={LOS}_{NI}*{DRR}_{NI}$$

We defined the difference between TRR and TRF as the opportunity costs of bed days used for NI patients. This means that we can rewrite equation (1) as:

4 $$OC={[LOS}_{NI}*{DRR}_{Normal}*OCR{]-[LOS}_{NI}*{DRR}_{NI}]$$

$$={LOS}_{NI}\left\{\left({DRR}_{Normal}*OCR\right)-{DRR}_{NI}\right\}$$

Equation (4) shows that the OC of bed days is determined by (i) the LOS for NI patients, (ii) the daily revenues from normal patients, (iii) the hospital or department's occupancy rate, and (iv) the daily revenues from NI patients. From the hospital standpoint, OC increases with longer NI patient stays, higher daily revenues from normal patients, and increased occupancy rates. Conversely, higher daily revenues from NI patients mitigate the OC associated with bed days.

### Statistical analysis

Due to the small nonrandom sample, we applied a matching algorithm whereby the generated p-values do not depend on sample size and prior knowledge of the correct propensity scores or the model to estimate them was not required [23–25]. We used genetic matching [23] for the group adjustment, as this matching method is nonparametric (unlike other common parametric preprocessing methods such as propensity score or Mahalanobis matching). Moreover, Genetic matching implements a search algorithm to maximize covariate balance [26, 27]. Nevertheless, we present estimates based on matching using propensity score (PSM), inverse probability weighting, Mahalanobis distance matching and generalized boosted modeling (see additional file 2). After genetic matching at a ratio of 1:3, we estimated the difference in LOS and daily revenue between patients with and without NI using a multivariate linear regression model. Statistical significance was defined as p < 0.05. Additionally, we conducted subgroup analyses for each covariate to identify potential cost drivers. All analyses were conducted using RStudio. Genetic matching was conducted using the R package “MatchIt” [28].

Covariates used in the genetic matching included age, sex, comorbidities measured by the American Society of Anesthesiologists (ASA) classification [29], and number of operations. The data were provided by the accounting department of the reporting hospital and checked for consistency. All costs were expressed in 2018 Euros. This study was approved by the ethics commission of the University Witten/Herdecke (Application No 215/2017 of February 08, 2018).

## Results

### Sample characteristics

The sample comprised 81 observations with NI and 2,324 observations without NI. Patients who spent less than one day in the hospital or who had missing data on relevant covariates were excluded from the study (see additional file 1).

Table 1 presents a comparison of the clinical and demographic attributes prior to and following matching. As shown in Table 1, the sample characteristics of baseline covariates were balanced between patients with NI and without NI using genetic matching. We achieved balance by examining the standardized mean difference and variance ratio (see additional file 3). Following matching, patients without NI had an average age of 55.8 years, while patients with NI had an average age of 57.0 years. Moreover, females made up 33.2% of patients without NI and 30.9% of patients with NI. In the non-infected control group, the average daily revenue was significantly lower for NI patients than for control patients (*p* < 0.001), while the average LOS per patient was significantly larger for NI patients (*p* < 0.001). The sample showed an unequal distribution of infection types since the data is a non-random sample from a reporting unit of a surgical and orthopedic hospital department. The most prevalent infection in the sample was SSI (77.88%), followed by CDAD (3.70%) and urinary tract infections (9.98%) (Table 2).

**Table 1:** Sample characteristics before and after matching

**Characteristics**	**Cases** **(*n* = 81)**	**Unmatched control Group** **(*n* = 2,324)**	***p*-value**	**Matched control Group** **(*n* = 209)**	***p*-value**
Age in years, median (IQR)	57.0 [42.0, 75.0]	52.0 [31.0, 65.0]	0.003^b^	55.0 [42.0, 71.0]	0.663^b^
No. of operations, n (%)					
One operation	60(74.1)	2181(93.8)	<0.001^a^	168(80.0)	0.415^a^
Two operations	8(9.9)	103(4.4)		20(9.5)	
>2 operations	13(16.0)	40(1.7)		22(10.5)	
Female, n (%)	25(30.9)	1,032(44.4)	0.017^a^	72(34.8)	0.581^a^
Comorbidity, n (%)					
Low comorbidity	15(18.5)	690(29.7)	<0.001^a^	40(19.0)	0.927^a^
Moderate Comorbidity	33(40.7)	1151(49.5)		90(42.9)	
High Comorbidity	33(40.7)	483(20.8)		80(38.1)	
Daily Revenue (€), median (IQR)	702.1 [497.7, 042.7]	999.1 [727.4,1,667.7]	<0.001^b^	957.80 [699.16, 1,605.51]	<0.001^b^
LOS, median (IQR)	19.0 [10.0, 28.0]	5.0 [3.0, 9.0]	<0.001b	7.0 [3.0, 13.0]	<0.001b

*SD* standard deviation, *LOS* Length of Stay, *IQR* Interquartile Range *P*-values were calculated using Fisher’s exact Test (a) and Mann-Whitney U test (b)

**Table 2:** Distribution of NI

**Type of NI**	**Number of cases**	**Frequency (%)**
UTI	8	9.88
SSI	63	77.78
CDAD	3	3.70
UTI+Penumonia	2	2.47
Pneumonia	2	2.47
Bronchitis	1	1.23
SSI+BSI	1	1.23
Thrombophlebitis	1	1.23

*UTI* urinary tract infection, *SSI* surgical site infection, *CDAD Clostridioides difficile* infectio*n*, *BSI* bloodstream infection

### Length of stay

After adjusting for baseline covariates through matching, patients with NIs exhibited a mean length of stay (LOS) of 19 days, in contrast to the 7 days observed for patients without NIs (*p* < 0.001). The multivariate model, which included confounding covariates, reaffirmed the estimates, with an estimated LOS of 10 days per patient (*p* < 0.001). We report the estimates from multivariate regression in Table 3. It is important to note that a longer LOS was also positively associated with more comorbidities. Patients with high comorbidities occupy approximately six additional hospital bed days compared to patients with low comorbidities. Similarly, patients who underwent two operations occupied approximately 13 additional bed days, and the number of occupied bed days more than tripled when patients underwent more than two operations (*p* < 0.001). However, after examining the remaining covariates, it was found that age (*p* = 0.121), sex (*p* = 0.378), and moderate comorbidity (*p* = 0.201) were not statistically significant predictors of LOS.

**Table 3:** Effect of NI on LOS, Daily Revenue and OC

	**Dependent Variable**		
**Variable**	**Length of Stay (LOS)**	**Daily Revenue**	**Opportunity Cost (OC)**
Infection	9.79 [1.87]^***^	-407.46 [105.21]^***^	NA
Age	0.08 [0.05]	-7.44 [2.78]^***^	-13.67 [43.43]
Gender[f]	-1.66 [1.88]	109.32 [105.47]	1001.60 [1690.93]
Moderate Comorbidity	3.21 [2.51]	6.52 [140.85]	4557.12 [2227.05]^**^
High Comorbidity	5.72 [2.71]^**^	-36.90 [152.46]	4136.75 [2415.15]
twice Operated	12.72 [2.99]^***^	-525.89 [168.18]^***^	8771.24 [2654.20]^***^
>2 times operated	32.71 [2.37]^***^	-473.62 [132.86]^***^	15920.42 [2100.38]^***^
Constant	-0.31 [2.90]	1764.52 [162.61]^***^	2272.64 [2476.24]
N	291	291	243
R2	0.480	0.144	0.239
F	37.315	6.784	12.348

Standard errors in square brackets; *p < 0.05, **p < 0.01, ***p < 0.001

The opportunity costs are estimated only for infected patients

### Daily revenue

Table 3 presents the results of the multivariate analysis with hospital daily revenue as the dependent variable. Notably, the presence of NI led to a reduction in daily revenue of more than €400 per day per patient (p < 0.001), which represents a 40% decrease from the median daily revenue earned by the hospital prior to the patient's infection. Unlike in the case of the LOS, the age of patients had a significant negative impact on daily revenues (*p* < 0.001). According to our multivariate estimates, every additional ten life-years reduce average daily revenue by more than €70 per day per patient.

In addition, a greater number of operations per patient was associated with lower daily revenue (*p* < 0.001). Since the reference group included patients who underwent a single operation, the number of operations may significantly predict the risk of infection [30]. Even among patients without NI, an increase in the number of operations (from one operation to two) reduced the median daily revenue by €266 and roughly tripled the median LOS from 5 to 14 days per patient. Therefore, patients without NI resemble those with such infections as the number of surgeries they undergo increases. In contrast, we found no significant associations between gender or comorbidities and daily revenue.

### Opportunity costs

The opportunity costs (OC) were calculated to determine the potential monetary losses for hospitals when treating patients with NI: OC represents the revenue forgone by the hospital due to the inability to treat additional, non-infected patients. Table 3 shows the linear regression of OC on baseline covariates. The findings indicate a statistically significant association between OC and both comorbidities and the number of operations. The number of operations was found to be a robust predictor of OC. Patients who underwent two operations had an OC of €8,725 (*p* < 0.001). Notably, patients who underwent more than two operations experienced more than double the OC, amounting to €16,105 (*p* < 0.001), compared with patients who underwent only a single operation.

We found a steady increase in the average LOS from 6 blocked bed days for a single operation to 17 days for two operations and further to 32 bed days for more than two operations. Remarkably, the daily revenue accrued to the hospital exhibited a contrasting trend: €1,351 for patients who underwent a single operation, €920 for those who underwent two operations, and €817 for patients who underwent more than two operations.

**Fig. 1:**
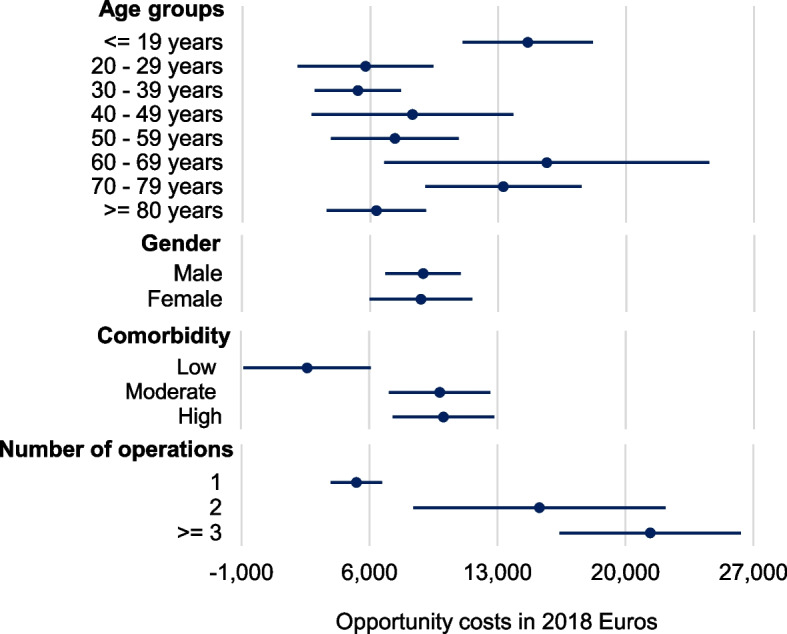
Subgroup analyses of opportunity costs

Analyzing opportunity costs across different subgroups gives an insight into associations between specific characteristics and the magnitude of opportunity costs (Figure 1). Across various age groups, the highest opportunity costs were observed among individuals under 19 years old, those aged 60 to 69 years, and those aged 70 to 79 years. Conversely, comparatively lower opportunity costs were identified in the age group of 30 to 39 years. Notably, there was minimal disparity in opportunity costs between men and women. Higher comorbidity levels appeared to correlate with increased opportunity costs, with patients exhibiting low comorbidity experiencing low opportunity costs compared to patients with moderate or high comorbidity.

Furthermore, the number of operations underwent by patients also influenced the magnitude of opportunity costs. Specifically, patients undergoing single operations were associated with lower opportunity costs, while those undergoing two or more operations exhibited higher opportunity costs.

## Discussion

The aim of this study was to estimate OC from a hospital perspective to determine the potential for infection prevention interventions in hospitals. Three main findings were reported. First, patients with NI stayed in the hospital significantly longer than patients without NI. Second, the daily revenue for patients with NI was significantly lower than that for patients without NI; third, the resulting OCs for patients with NI are high and pose a substantial economic burden from a hospital perspective. Therefore, we see strong economic incentives for hospitals to implement appropriate infection prevention measures.

Our findings are in line with those of previous studies. An article from Germany considering only CDAD estimated the opportunity cost to be €5,263 [8]. The same applies to the additional LOS in patients with NI. The published literature reports an excess LOS of approximately 10 days for patients with SSI [9, 31]. In a related study, Eckmann et al. [22] found that patients with SSIs experienced a hospital stay that extended 16 days longer than their control patients. This higher estimate may be attributed to issues with data comparability among various healthcare facilities or the challenges of accurately tracking recurrent SSIs. In contrast, our routine data provides a more reliable means of monitoring recurring infections within a single hospital.

We found that comorbidities had a significant effect on the LOS. Previous studies have shown that comorbidities contribute to a longer LOS [32, 33]. For patients with multiple comorbidities, there might also be a greater risk of developing (more) complications and being less independent than for patients with fewer comorbidities. Both aspects lead to longer hospital stays, which then increase the risk of NI [30]. For example, Karaoui et al. [34] identified previous hospitalization and comorbidities as significant risk factors for NI. A similar observation applies when looking at the number of operations. Obviously, the more frequently a patient undergoes surgery, the longer the LOS and the greater the risk that this patient will acquire an NI. Longer hospital stays can lead to colonization of patients with pathogenic microorganisms, increasing the likelihood of infection with NI [30, 35, 36]. Similarly, we found that both comorbidity and the number of operations were statistically significant predictors (*p* < 0.001) of prolonged LOS in our multivariate regression model.

In addition to prolonging the LOS, NIs impose a significant economic burden on hospitals because they result in a loss in daily revenues. The multivariate regression underscores that age serves as a statistically significant predictor of the loss in daily hospital revenues. The estimated coefficient implies that each additional 10 life-years reduced daily revenue by approximately €70. This finding aligns with the observations of Lange et al. [37], who found that the main cost drivers for high-cost insureds in the German hospital sector were cerebral infarction, heart failure, atherosclerosis, fracture of the femur, and acute myocardial infarction. These diseases, which are more common in older people, may explain the significant effect of age on daily revenues.

Our analysis aims to shed light on the economic incentives for preventing NIs from a hospital’s perspective. The results indicate that individuals with NIs are associated with a daily revenue decline surpassing €400 per patient. Given our robust estimate of an additional 10-day LOS due to NI, the estimated daily revenue loss translates to an approximate revenue loss of €4,000 per NI patient. Our estimate is in line with the assessment of revenue loss for CDAD in Germany by Grube et al. [21], who calculated losses of €3,442 for secondary diagnoses and €4,194 for recurrent diagnoses using the cost data collected under the Hospital Remuneration Act (Krankenhausentgeltgesetz, KHEntgG) §21[20] from 2011. Similarly, Eckmann et al. [22] used the same nationally collected data from 2010 to 2016 and found comparable results related to SSIs, indicating that these infections incur additional costs of around €10,000 per case.

Overall, our findings suggest that hospitals can maintain profitability by effectively managing NIs, as they have the potential to replace each NI patient with 2 non-NI patients, thereby increasing overall hospital gross revenues [38]. Assuming that most of a hospital´s costs are fixed (e.g., staff costs), this also means higher profits for the hospital.

The multivariate analysis underscores the substantial impact of comorbidity and the number of operations on the hospital's OC. Specifically, patients with baseline comorbidities experience prolonged hospital stays, leading to a notable increase in forgone revenue and, consequently, OC. These findings align with those of Karaoui et al. [25], who highlighted the significance of prior comorbidity scores as primary predictors in their cohort study. Additionally, the number of operations has emerged as a pivotal factor influencing OC for patients with NI. From a hospital perspective, managing and minimising comorbidities and optimising surgical procedures can significantly contribute to reducing the OC associated with treating NI patients.

At the same time, given an average OCR of 81.6% for Germany in 2016, the estimates of LOS and daily revenue suggest that preventing a single NI case would lead to the release of 10 blocked hospital beds per NI patient, potentially allowing 2 additional admissions of non-NI patients (median LOS). Since the NI patient pays €700 (median DRG for NI patients) and the non-NI patients each pay €1,000, preventing a single NI would be equivalent to an increase in gross daily revenues for the hospital of €1,300. Since 30-50% of NIs are preventable [39], these estimates imply that hospital revenue increases by approximately €390 to €650 per NI patient prevented per day. If the hospital has no extra non-NI patient to fill the newly freed beds, the loss of revenue often leads to changes in resource allocation and/or staffing [38].

### Strength and limitations

This study analysed the economic burden of NIs on a surgical and orthopedic unit of a German hospital from 2018 to 2019 using data provided by routine accounting department data. While our study provides valuable insights into the economic evaluation of NIs, it is important to acknowledge certain limitations. We lacked information on some sociodemographic variables, such as patients' education and income levels. Therefore, our multivariate regression estimates may be biased due to unobserved and unadjusted covariates via channels related to healthcare practices and access to treatments.

Furthermore, the type of infection was not evenly distributed across the sample, and SSI was the most common infection, as the data were collected from a sample of patients treated at an orthopedic or surgical unit. As a result, we were unable to analyse the revenue loss, LOS and OC per infection type. However, our results have a high transferability for similar units in other hospitals. Nevertheless, to the best of our knowledge, this is the first study estimating opportunity costs in Germany considering different types of NI. The applied matching method enabled us to generate a good balanced sample and thus estimate robust outcomes. Therefore, the findings of this study could provide valuable input for future economic assessments.

### Conclusion and implications

NIs are associated with high opportunity costs for hospitals. Therefore, it can be concluded that the economic benefits of preventing NI are high. Hospitals have strong incentives to implement comprehensive infection control measures and interventions. Currently, there is evidence supporting the effectiveness of various measures for preventing NI. These measures include implementing a complex infection prevention bundle by a prevention link physician [40], introducing an infection control link nurse [41], surveilling the NI [42], improving adherence to hand hygiene [40, 43] and changing dressing. A next step should be to evaluate the cost-effectiveness of these interventions in Germany.

## Supplementary Information

Additional file 1: Supplementary Table 1 Excluded missing values Description of why and how many observations we dropped due to missingness.

Additional file 2: Supplementary Table 2 Treatment effect estimates using alternative models. Supplementary treatment effect estimation using alternative matching models other than genetic matching.

Additional file 3: Supplementary figure 1 Covariate balance after genetic matching. Display of covariate balance using standardized mean difference (SMD) and variance ratio between patients with NI and those without.

Additional file 4: Supplementary Table 3 Effect of NI on LOS, Daily Revenue and OC. An extended version of the main Table 3 for further inference.

Additional file 5.

## Data Availability

The data that support the findings of this study are available on request from the corresponding author. The data are not publicly available due to privacy or ethical restrictions.

## Abbreviations

NI: Nosocomial Infection
OCR: Occupancy Rate
SSI: Surgical Site Infection
CDAD: *Clostridium Difficile* Associated Diarrhea
TRF: Total Revenue Forgone
TRR: Total Realized Revenue
DRR: Daily Realized Revenue
OC: Opportunity Cost
PSM: Propensity Score Matching
UTI: Urinary Tract Infection
BSI: Bloodstream Infection
