# Supplementary material for: The economic burden of nosocomial infections for hospitals: evidence from Germany
Source: BMC Infect Dis. 2024 Nov 13;24:1294. doi: 10.1186/s12879-024-10176-8 (PMC11562106; doi:10.1186/s12879-024-10176-8)
Supplement: Supplementary file 1 — Additional file 1: Supplementary Table 1 Excluded missing values Description of why and how many observations we dropped due to missingness. [file 12879_2024_10176_MOESM1_ESM.docx]

| **Supplementary Table 1:** Excluded missing values | |
| --- | --- |
| Reason for excluding observations | No. of observations excluded (n = 188) |
| Patients with 0 hospital stays | 78 |
| Missing values in LOS | 26 |
| Missing values in no. of operations | 4 |
| Missing values in gender | 0 |
| Missing values on infection | 2 |
| Missing values in treated body part | 3 |
| Missing values in ASA score | 82 |

LOS=Length of Stay; ASA score= American Society of Anaesthesiologists
