# Supplementary material for: The economic burden of nosocomial infections for hospitals: evidence from Germany
Source: BMC Infect Dis. 2024 Nov 13;24:1294. doi: 10.1186/s12879-024-10176-8 (PMC11562106; doi:10.1186/s12879-024-10176-8)
Supplement: Supplementary file 2 — Additional file 2: Supplementary Table 2 Treatment effect estimates using alternative models. Supplementary treatment effect estimation using alternative matching models other than genetic matching. [file 12879_2024_10176_MOESM2_ESM.docx]

| **Supplementary Table 2** Treatment effect estimates using alternative models | | | | |
| --- | --- | --- | --- | --- |
| **a. Dependent Variable: LOS** | | | | |
|  | IPW | PSM | GBM | MDM |
| Age | 0.08 | 0.12 | 0.06 | 0.05 |
|  | [0.01]*** | [0.04]*** | [0.01]*** | [0.06] |
| Infection | 9.57 | 10.89 | 9.18 | 9.19 |
|  | [0.49]*** | [1.65]*** | [0.49]*** | [2.06]*** |
| Gender(f) | -0.47 | -2.99 | -1.06 | -0.07 |
|  | [0.41] | [1.67]* | [0.50]** | [2.15] |
| Moderate Comorbidity | 1.18 | 1.19 | 2.79 | 1.46 |
|  | [0.50]** | [2.16] | [0.62]*** | [2.80] |
| High Comorbidity | 3.24 | 1.53 | 5.64 | 4.41 |
|  | [0.66]*** | [2.33] | [0.77]*** | [3.04] |
| Two Operations | 11.46 | 8.60 | 12.66 | 12.84 |
|  | [0.83]*** | [2.96]*** | [0.96]*** | [3.34]*** |
| >2 Operations | 28.26 | 29.01 | 31.04 | 35.04 |
|  | [1.11]*** | [2.09]*** | [1.04]*** | [2.66]*** |
| Constant | 1.61 | -0.01 | 1.41 | 2.15 |
|  | [0.55]*** | [2.66] | [0.70]** | [3.25] |
| N | 2405 | 294 | 2405 | 280 |
| R2 | 0.41 | 0.50 | 0.46 | 0.45 |
| F | 242.16 | 40.47 | 296.82 | 31.99 |
| **b. Dependent Variable: Daily Revenue** | | | | |
|  | IPW | PSM | GBM | MDM |
| Age | -4.37 | -7.48 | -1.60 | -6.50 |
|  | [1.05]*** | [2.82]*** | [0.93]* | [2.75]** |
| Infection | -363.08 | -430.87 | -385.13 | -372.28 |
|  | [44.76]*** | [104.87]*** | [33.72]*** | [101.26]*** |
| Gender(f) | -36.79 | 81.74 | -71.20 | 181.22 |
|  | [37.29] | [105.84] | [34.17]** | [105.42]* |
| Moderate Comorbidity | -200.15 | -32.17 | -242.27 | -117.66 |
|  | [46.01]*** | [136.63] | [42.40]*** | [137.28] |
| High Comorbidity | -257.82 | -57.52 | -287.90 | -128.46 |
|  | [60.76]*** | [147.48] | [52.97]*** | [149.13] |
| Two Operations | -369.90 | -488.18 | -346.88 | -506.88 |
|  | [75.76]*** | [187.28]*** | [65.61]*** | [163.78]*** |
| >2 Operations | -448.11 | -462.48 | -362.59 | -486.41 |
|  | [101.90]*** | [132.55]*** | [71.46]*** | [130.51]*** |
| Constant | 1733.02 | 1822.03 | 1635.66 | 1746.16 |
|  | [50.43]*** | [168.48]*** | [47.86]*** | [159.19]*** |
| N | 2405 | 294 | 2405 | 280 |
| R2 | 0.10 | 0.15 | 0.13 | 0.16 |
| F | 35.95 | 6.93 | 49.78 | 7.15 |

**IPW**=Inverse Propensity score weighting; **PSM**=Propensity Score Matching; **GBM**=Generalized boosted modeling or gradient boosting machines; **MDM**=Mahalanobis Distance Matching. Standard errors in square brackets; * p < 0.05, ** p < 0.01, *** p < 0.001
