# Supplementary material for: The economic burden of nosocomial infections for hospitals: evidence from Germany
Source: BMC Infect Dis. 2024 Nov 13;24:1294. doi: 10.1186/s12879-024-10176-8 (PMC11562106; doi:10.1186/s12879-024-10176-8)
Supplement: Supplementary file 3 — Additional file 3: Supplementary figure 1 Covariate balance after genetic matching. Display of covariate balance using standardized mean difference (SMD) and variance ratio between patients with NI and those without. [file 12879_2024_10176_MOESM3_ESM.pdf]

Supplementary Figure 1 Covariate Balance after Genetic Matching

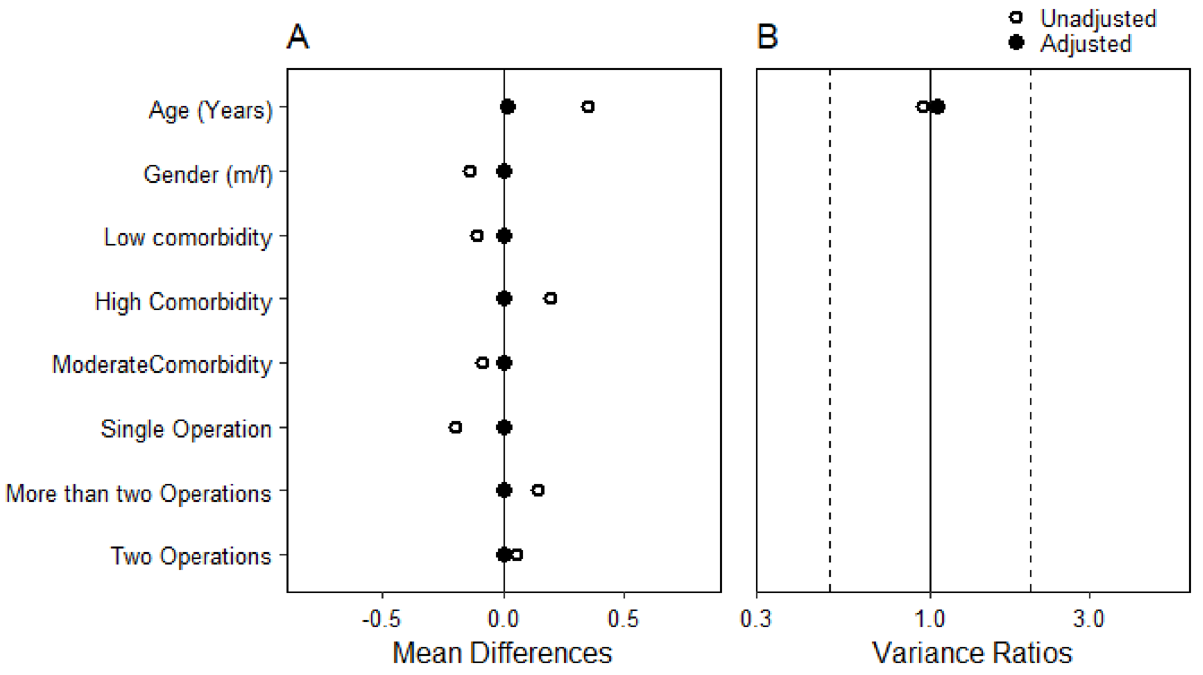

Panel A: Dot plot showing the success of genetic matching for the standardized means of key covariates. The open circles represent the standardized mean difference between the unmatched NI and control groups at baseline. The dark-filled circles represent the standardized mean difference between the matched NI and control groups at baseline. The initial large differences are reduced to near 0 through genetic matching. Panel B: The open circles represent the ratio of the variance of the NI to the control group (non-NI) at baseline. The dark-filled circles represent the ratio of the variances of the matched NI and control groups at baseline. The initial differences in the variance ratios in age are reduced to near 1 (equal variances) through genetic matching
