# Supplementary material for: The economic burden of nosocomial infections for hospitals: evidence from Germany
Source: BMC Infect Dis. 2024 Nov 13;24:1294. doi: 10.1186/s12879-024-10176-8 (PMC11562106; doi:10.1186/s12879-024-10176-8)
Supplement: Supplementary file 4 — Additional file 4: Supplementary Table 3 Effect of NI on LOS, Daily Revenue and OC. An extended version of the main Table 3 for further inference. [file 12879_2024_10176_MOESM4_ESM.docx]

| **Supplementary Table 3** Effect of NI on LOS, Daily Revenue and OC | | | | | | |
| --- | --- | --- | --- | --- | --- | --- |
| **a. Length of Stay (LOS)** | | | | | | |
| Variable | **(1)** | [SE] | **(2)** | [SE] | **(3)** | [SE] |
| Age | 0.17 | [0.06]*** | 0.07 | [0.06] | 0.08 | [0.05] |
| Infection | 9.79 | [2.47]*** | 9.77 | [2.44]*** | 9.79 | [1.87]*** |
| Gender(f) | -4.89 | [2.43]** | -5.05 | [2.42]** | -1.66 | [1.88] |
| Moderate Comorbidity |  |  | 5.22 | [3.15]* | 3.21 | [2.51] |
| High Comorbidity |  |  | 10.47 | [3.50]*** | 5.72 | [2.71]** |
| twice Operated |  |  |  |  | 12.72 | [2.99]*** |
| >2 times operated |  |  |  |  | 32.71 | [2.37]*** |
| Constant | 5.75 | [3.39]* | 4.93 | [3.73] | -0.31 | [2.90] |
| N | 291 |  | 291 |  | 291 |  |
| R2 | 0.085 |  | 0.114 |  | 0.480 |  |
| F | 8.906 |  | 7.332 |  | 37.315 |  |
| **b. Daily Revenue** | | | | | | |
| Variable | **(1)** | [SE] | **(2)** | [SE] | **(3)** | [SE] |
| Age | -7.84 | [2.46]*** | -6.85 | [2.86]** | -7.44 | [2.78]*** |
| Infection | -407.37 | [108.31]*** | -407.22 | [108.55]*** | -407.46 | [105.21]*** |
| Gender(f) | 145.03 | [106.77] | 150.88 | [107.73] | 109.32 | [105.47] |
| Moderate Comorbidity |  |  | -98.52 | [140.16] | 6.52 | [140.85] |
| High Comorbidity |  |  | -127.51 | [155.92] | -36.90 | [152.46] |
| twice Operated |  |  |  |  | -525.89 | [168.18]*** |
| >2 times operated |  |  |  |  | -473.62 | [132.86]*** |
| Constant | 1636.19 | [149.00]*** | 1669.86 | [166.30]*** | 1764.52 | [162.61]*** |
| N | 291 |  | 291 |  | 291 |  |
| R2 | 0.080 |  | 0.082 |  | 0.144 |  |
| F | 8.285 |  | 5.090 |  | 6.784 |  |
| **c. Opportunity Cost (OC)** | | | | | | |
| Variable | **(1)** | [SE] | **(2)** | [SE] | **(3)** | [SE] |
| Age | 27.17 | [42.41] | -19.23 | [48.47] | -13.67 | [43.43] |
| Gender(f) | -365.48 | [1880.09] | -871.84 | [1870.86] | 1001.60 | [1690.93] |
| Moderate Comorbidity |  |  | 6177.92 | [2409.50]** | 4557.12 | [2227.05]** |
| High Comorbidity |  |  | 6724.07 | [2685.13] | 4136.75 | [2415.15] |
| Two Operations |  |  |  |  | 8771.24 | [2654.20]*** |
| >2 Operations |  |  |  |  | 15920.42 | [2100.38]*** |
| Constant | 7339.66 | [2485.31]*** | 4873.49 | [2754.54]* | 2272.64 | [2476.24] |
| N | 243 |  | 243 |  | 243 |  |
| R2 | 0.002 |  | 0.033 |  | 0.239 |  |
| F | 0.206 |  | 2.003 |  | 12.348 |  |

Model (1) considers age & gender as covariates. Model (2) further includes comorbidity and in model (3), we consider the number of operations as additional factor. Panel (a) presents OLS regression of LOS, panel (b) presents the OLS regression of hospital daily revenue, and panel (b) presents OLS regression of OC. SE = standard error; * p < 0.05, ** p < 0.01, *** p < 0.001
