# Supplementary figures and images for: The economic burden of nosocomial infections for hospitals: evidence from Germany
Source: BMC Infect Dis. 2024 Nov 13;24:1294. doi: 10.1186/s12879-024-10176-8 (PMC11562106; doi:10.1186/s12879-024-10176-8)

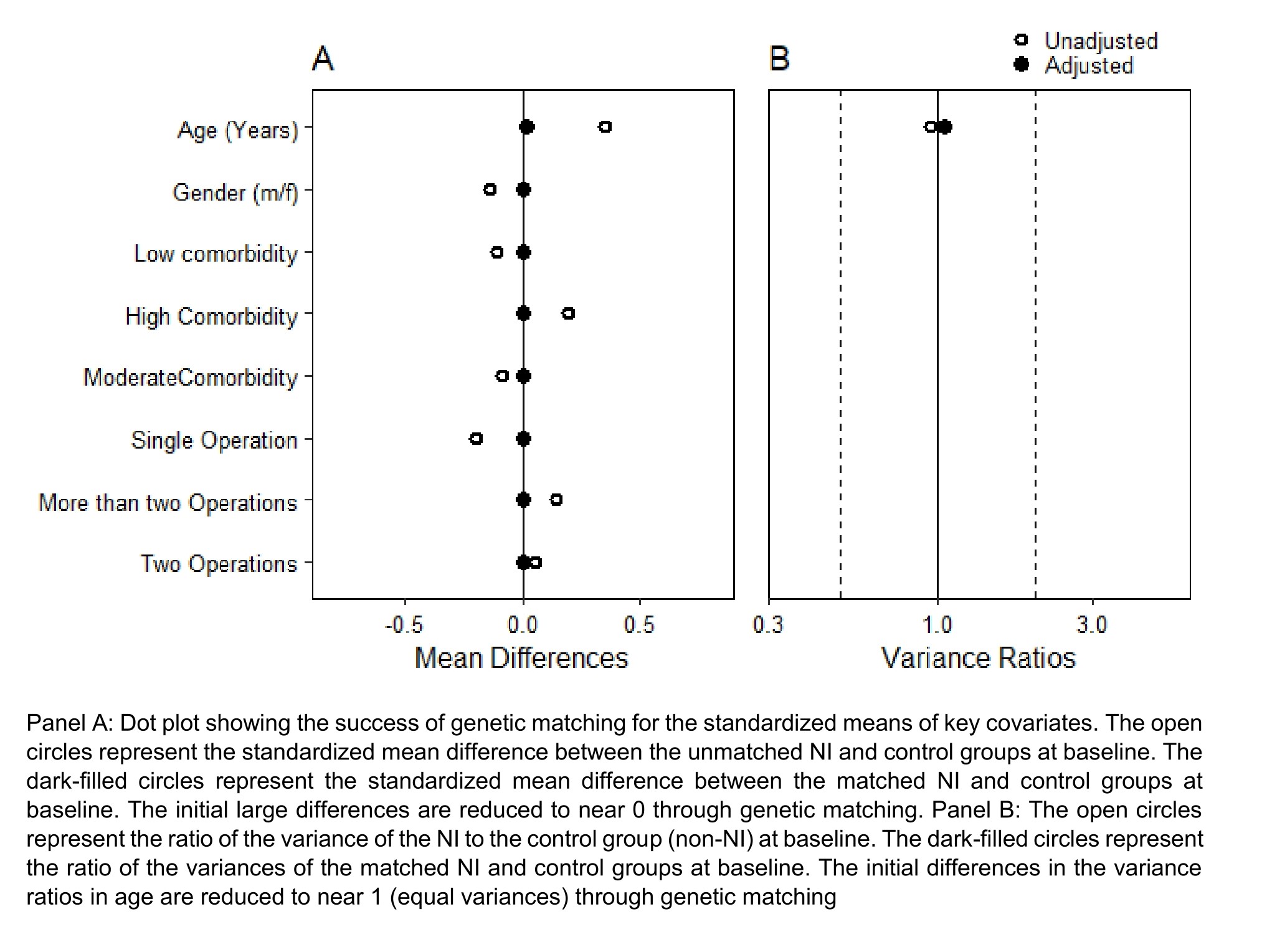

Supplement: Supplementary file 5 — Additional file 5. [file 12879_2024_10176_MOESM5_ESM.jpg]
